# Tissue Cytomegalovirus DNA Detection by PCR Is Associated with Increased Endoscopic Activity in Ulcerative Colitis: A Retrospective Cohort Study

**DOI:** 10.3390/biomedicines14020461

**Published:** 2026-02-19

**Authors:** Omer Kucukdemirci, Hasan Eruzun, Berk Bas, Muge Ustaoglu, Ufuk Avcioglu, Elfag Isgandarov, Ahmet Bektas

**Affiliations:** 1 Department of Gastroenterology and Hepatology, Hakkari State Hospital, 30000 Hakkari, Türkiye; 2 Department of Gastroenterology and Hepatology, Samsun Training and Research Hospital, 55100 Samsun, Türkiye; 3 Department of Gastroenterology and Hepatology, School of Medicine, Aydın Adnan Menderes University, 09100 Aydın, Türkiye; berkcorn@gmail.com; 4 Department of Gastroenterology and Hepatology, School of Medicine, Ondokuz Mayis University, 55100 Samsun, Türkiye; ustaoglu.md@gmail.com (M.U.); ufukavcioglu@yahoo.com (U.A.); dr.elfaq@gmail.com (E.I.); abektas@omu.edu.tr (A.B.)

**Keywords:** cytomegalovirus, ulcerative colitis, endoscopic activity, Rachmilewitz index, Mayo score, PCR, steroid use

## Abstract

**Background:** Cytomegalovirus (CMV) reactivation is frequently observed in patients with moderate-to-severe ulcerative colitis (UC), particularly in steroid-refractory cases; however, the clinical significance of tissue CMV DNA detection by polymerase chain reaction (PCR) in the absence of classical histopathological findings remains controversial. **Methods:** This retrospective cohort study included 110 patients with moderate-to-severe UC who underwent colonoscopy between January 2021 and April 2024 at a tertiary referral center. Tissue and serum samples were analyzed for CMV DNA using PCR. Tissue CMV DNA positivity was defined as ≥250 copies. Endoscopic disease activity was assessed using the Mayo clinical score, Mayo endoscopic score, and the Rachmilewitz Endoscopic Activity Index. Associations between tissue CMV DNA positivity, endoscopic activity scores, inflammatory markers, recent immunosuppressive therapy, and serum CMV PCR results were evaluated. Sensitivity analyses using different tissue CMV DNA thresholds and receiver operating characteristic (ROC) curve analysis for serum CMV PCR were also performed. **Results:** Tissue CMV DNA positivity was detected in 37.3% of patients. Patients with tissue CMV DNA positivity had significantly higher Mayo clinical scores and Rachmilewitz Endoscopic Activity Index scores compared with CMV-negative patients, whereas Mayo endoscopic scores did not differ significantly between groups. Serum CMV PCR levels were markedly higher in patients with tissue CMV DNA positivity (*p* < 0.001). Tissue CMV DNA copy number showed a strong correlation with serum CMV PCR levels but did not demonstrate a consistent linear association with endoscopic activity scores. In multivariable logistic regression analysis, recent corticosteroid use was independently associated with tissue CMV DNA positivity, while anti-TNF therapy and endoscopic activity indices were not independent predictors. ROC analysis demonstrated good diagnostic performance of serum CMV PCR for predicting tissue CMV DNA positivity (AUC = 0.82). **Conclusions:** In patients with moderate-to-severe UC, tissue CMV DNA positivity (≥250 copies) is associated with increased clinical and endoscopic disease activity, even in the absence of classical histopathological evidence of CMV infection. These findings suggest that molecular detection of CMV in colonic tissue may provide clinically relevant information in selected patient populations, particularly those with recent corticosteroid exposure. However, tissue CMV DNA positivity should be interpreted as a molecular association rather than definitive evidence of causality or an indication for antiviral therapy. Prospective multicenter studies are warranted to further clarify the clinical implications of tissue CMV DNA detection in UC.

## 1. Introduction

Cytomegalovirus (CMV), a double-stranded DNA virus from the Herpesviridae family, can present asymptomatically, with systemic symptoms, or as tissue-invasive disease. The gastrointestinal tract is frequently affected, accounting for 30% of tissue-invasive cases in immunocompetent patients [1]. A high prevalence of CMV infection has been identified in patients with ulcerative colitis, particularly in severe corticosteroid-refractory cases, where the infection rate ranges from 20% to 40% based on diagnoses made using antigenemia and histological examination of tissue biopsies [2,3]. In immunocompetent individuals, CMV colitis is predominantly asymptomatic or manifests as a self-limiting condition [1,4]. However, it can potentially progress to a chronic infection or establish a lifelong carrier state characterized by intermittent viral reactivation [5]. CMV reactivation is commonly observed in cases of severe ulcerative colitis or patients exhibiting resistance to corticosteroid therapy. Nonetheless, it remains unclear whether CMV plays a causative role in exacerbating ulcerative colitis or merely represents an incidental finding associated with severe disease states [6,7]. On the other hand, some studies have suggested that there may be a close relationship between inflammation promoting viral expression and viral replication exacerbating inflammation [8]. The optimal approach for diagnosing CMV colitis remains unclear; however, Haematoxylin and Eosin (H&E) staining, immunohistochemistry (IHC), and tissue PCR are considered the preferred methods for detecting CMV in colonic tissue. These techniques are deemed more reliable for assessing CMV involvement in colitis than antigenemia testing, IgM serology, or CMV DNA PCR analysis [7,9]. Conversely, the delayed results of methods such as H&E staining in patients with acute severe ulcerative colitis may postpone the initiation of treatment for CMV-complicated colitis, potentially leading to serious complications [10,11]. Therefore, the European Crohn’s and Colitis Organization (ECCO) guidelines recommend initiating of antiviral therapy in patients identified with CMV reactivation [12]. The Mayo Scoring System and the Rachmilewitz Index are commonly used tools for assessing the activity of UC [13,14]. These indices help in evaluating disease severity and guiding treatment decisions [15,16]. Some studies, albeit indirectly, have demonstrated that histologically confirmed CMV infection in colonic tissue is associated with increased inflammatory activity and elevated endoscopic activity scores in patients with UC [3,6,17,18]. However, there is a limited number of studies that examine the direct impact of CMV detection through PCR in tissue samples on inflammatory and endoscopic severity, without correlating these findings with IHC or the presence of CMV inclusion bodies. This study aims to compare the quantitative levels of CMV DNA, measured via PCR in tissue samples from ulcerative colitis patients experiencing moderate to severe flares—classified according to the Truelove and Witts criteria—with their corresponding endoscopic activity scores. Furthermore, we seek to evaluate the potential correlation between the presence of CMV and disease severity in this context.

## 2. Materials and Methods

### 2.1. Study Design and Ethical Approval

This study was designed as a single-center, retrospective cohort study conducted at Ondokuz Mayıs University Faculty of Medicine, Department of Gastroenterology and Hepatology. The study protocol was reviewed and approved by the Ondokuz Mayıs University Clinical Research Ethics Committee on 4 April 2024 (approval number: 2024000169-1; file number: 2024/169). Due to the retrospective nature of the study, informed consent was waived in accordance with institutional regulations and national ethical guidelines.

### 2.2. Patient Selection

Between January 2021 and April 2024, a total of 231 patients diagnosed with moderate-to-severe ulcerative colitis according to the Truelove and Witts criteria were evaluated at our center. Among these, 110 patients who underwent colonoscopy or flexible rectosigmoidoscopy during disease exacerbation and had both tissue and serum samples obtained for cytomegalovirus (CMV) analysis were included in the study.

**Figure 1:**
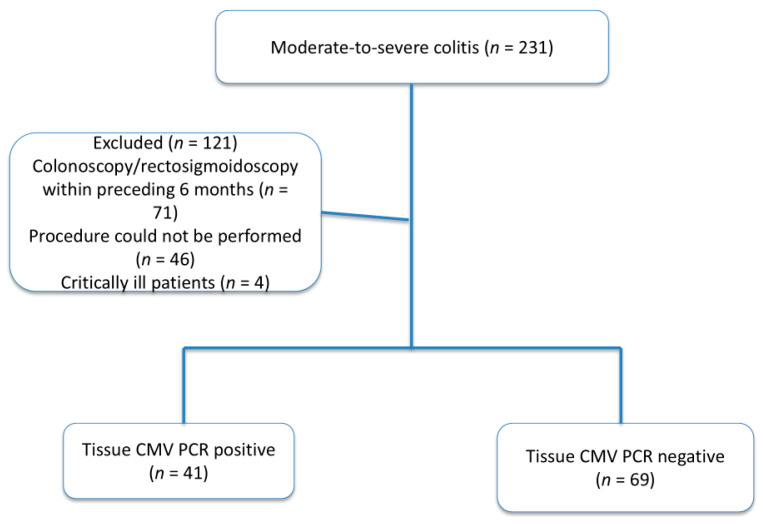
Flowchart of the participants. CMV PCR: cytomegalovirus polymerase chain reaction. A total of 231 patients were assessed for the study; 121 were excluded, and 110 were included.

Patients were excluded if they had undergone endoscopic evaluation within the preceding six months to prevent redundancy, if endoscopic assessment could not be performed because of technical constraints or clinical instability, or if they were critically ill requiring immediate intensive medical management. Critically ill patients were excluded to ensure patient safety and methodological consistency, as endoscopic evaluation and tissue biopsy necessary for tissue CMV PCR analysis could not be performed safely or reliably. A detailed flowchart illustrating patient selection and exclusion criteria is provided in Figure 1.

Demographic data, disease duration, follow-up time, laboratory parameters, stool frequency, endoscopic activity scores, serum CMV PCR results, histopathological findings, and tissue CMV PCR copy numbers were retrospectively collected from electronic medical records.

### 2.3. Endoscopic Evaluation and Tissue Sampling

All endoscopic procedures were performed using a standard colonoscope (Fujinon EC-760R; Fujifilm Medical Systems, Tokyo, Japan) by experienced gastroenterologists with a minimum of two years of independent endoscopy practice. Endoscopic disease activity was assessed during the procedure using both the Modified Mayo Endoscopic Score and the Rachmilewitz Endoscopic Activity Index (Table 1).

For CMV PCR analysis, a single mucosal biopsy specimen sufficient to fill the biopsy forceps was obtained from inflamed colonic segments using standard disposable forceps (Disposable biopsy forceps, 2.8 mm; Micro-Tech Endoscopy, Nanjing, China). Tissue samples were immediately placed in sterile saline and transported under appropriate conditions to the microbiology laboratory. Additional biopsy samples from the same region were collected for routine histopathological evaluation and immunohistochemical analysis and were fixed in 4% formalin.

### 2.4. CMV PCR Analysis and Definition of Tissue CMV DNA Positivity

Quantitative detection of CMV DNA in both tissue and serum samples was performed using real-time polymerase chain reaction (RT-PCR) in the hospital’s central microbiology laboratory, following standardized laboratory protocols.

Based on previously published studies and institutional laboratory validation data, tissue CMV DNA positivity was defined as a viral load of ≥250 copies per sample [19]. This threshold was selected to distinguish clinically relevant CMV DNA detection from low-level viral presence that may represent latent infection or contamination. Patients were classified into CMV DNA-positive and CMV DNA-negative groups according to this predefined cut-off value.

Importantly, tissue CMV DNA positivity was evaluated independently of histopathological findings, and patients were not classified as having CMV colitis unless classical histological features or immunohistochemical positivity were present. For the purposes of the present study, tissue CMV DNA positivity was treated as an analytical classification rather than a clinical diagnosis, and antiviral therapy decisions were based exclusively on histopathological or immunohistochemical confirmation of CMV infection.

### 2.5. Statistical Analysis

Patients were categorized into two groups based on tissue CMV PCR results (CMV DNA-positive vs. CMV DNA-negative). Continuous variables were assessed for normality using the Kolmogorov–Smirnov test. Normally distributed variables were compared using independent sample *t*-tests, while non-parametric variables were analyzed using the Mann–Whitney U test. Categorical variables were compared using Pearson’s chi-square test.

Correlation analyses between tissue CMV DNA copy number and clinical, laboratory, and endoscopic parameters were performed using Spearman’s rank correlation coefficient. Multivariable logistic regression analyses were conducted to identify independent predictors of tissue CMV DNA positivity, including demographic variables, recent corticosteroid use, anti-TNF therapy, and endoscopic activity indices.

To evaluate the robustness of the predefined tissue CMV DNA cut-off value, sensitivity analyses were performed using alternative thresholds (≥100, ≥250, ≥500, and ≥1000 copies). The diagnostic performance of serum CMV PCR for predicting tissue CMV DNA positivity was assessed using receiver operating characteristic (ROC) curve analysis, with calculation of the area under the curve (AUC) and determination of the optimal cut-off value using the Youden index. Statistical significance was defined as *p* < 0.05. All analyses were performed using SPSS software version 26 (IBM Corp., Armonk, NY, USA).

## 3. Results

A total of 110 patients with moderate-to-severe ulcerative colitis were included in the study. Based on tissue CMV PCR results, 41 patients (37.3%) were classified as tissue CMV DNA-positive (≥250 copies), while 69 patients (62.7%) were classified as CMV DNA-negative. All patients underwent endoscopic evaluation during an active disease flare, and tissue and serum samples were obtained concurrently.

Baseline demographic, clinical, laboratory, and endoscopic characteristics of patients with and without tissue CMV DNA positivity are presented in Table 2. There were no statistically significant differences between the two groups with respect to age, sex distribution, follow-up duration, white blood cell count, C-reactive protein levels, erythrocyte sedimentation rate, or daily defecation frequency (all *p* > 0.05). In contrast, serum CMV PCR levels were significantly higher in patients with tissue CMV DNA positivity compared with CMV-negative patients (mean ± SD: 1104.3 ± 2520.7 vs. 18.3 ± 66.5 copies; median: 57 vs. 0 copies; *p* < 0.001).

With regard to disease activity, patients with tissue CMV DNA positivity exhibited higher endoscopic and clinical activity scores. The Rachmilewitz Endoscopic Activity Index was significantly higher in the CMV-positive group (9.0 ± 2.3 vs. 7.8 ± 2.5; *p* = 0.021). Similarly, the Mayo clinical score was significantly increased in patients with tissue CMV DNA positivity (8.7 ± 2.5 vs. 7.6 ± 2.4; *p* = 0.014). In contrast, Mayo endoscopic scores did not differ significantly between CMV-positive and CMV-negative patients (2.5 ± 0.5 vs. 2.3 ± 0.6; *p* = 0.161).

Correlation analyses evaluating the relationship between tissue CMV DNA copy number and clinical, laboratory, and endoscopic parameters are shown in Table 3. Tissue CMV DNA copy number demonstrated a strong positive correlation with serum CMV PCR levels (Spearman r = 0.595, *p* < 0.001). No significant correlations were observed between tissue CMV DNA copy number and age, follow-up duration, inflammatory markers (CRP or ESR), daily defecation frequency, Mayo clinical score, or Mayo endoscopic score (all *p* > 0.05). A borderline positive association was observed between tissue CMV DNA copy number and the Rachmilewitz Endoscopic Activity Index (r = 0.179, *p* = 0.063).

Multivariable logistic regression analyses were performed to identify independent predictors of tissue CMV DNA positivity (≥250 copies), and the results are summarized in Table 4. In the clinical risk model, recent corticosteroid use within the preceding three months was independently associated with tissue CMV DNA positivity (odds ratio [OR] 2.58, 95% confidence interval [CI] 1.01–6.62; *p* = 0.048). Age, sex, follow-up duration, and anti-TNF therapy were not independently associated with tissue CMV DNA positivity. In the disease activity–adjusted model, corticosteroid use retained a positive association with tissue CMV DNA positivity, although this association did not reach statistical significance (OR 2.32, 95% CI 0.92–5.85; *p* = 0.076). The Rachmilewitz Endoscopic Activity Index was not an independent predictor of tissue CMV DNA positivity in this model.

Sensitivity analyses using different tissue CMV DNA thresholds (≥100, ≥250, ≥500, and ≥1000 copies) are presented in Table 5. Across all thresholds, neither Mayo clinical scores nor Rachmilewitz Endoscopic Activity Index scores demonstrated a consistent or statistically significant dose–response relationship with increasing tissue CMV DNA levels. These findings suggest the presence of a threshold effect rather than a linear association between CMV viral burden and disease activity. Only a small number of patients had very high tissue CMV DNA levels (≥5000 copies, *n* = 7; ≥10000 copies, *n* = 4), precluding meaningful statistical analysis at these higher thresholds.

**Figure 2:**
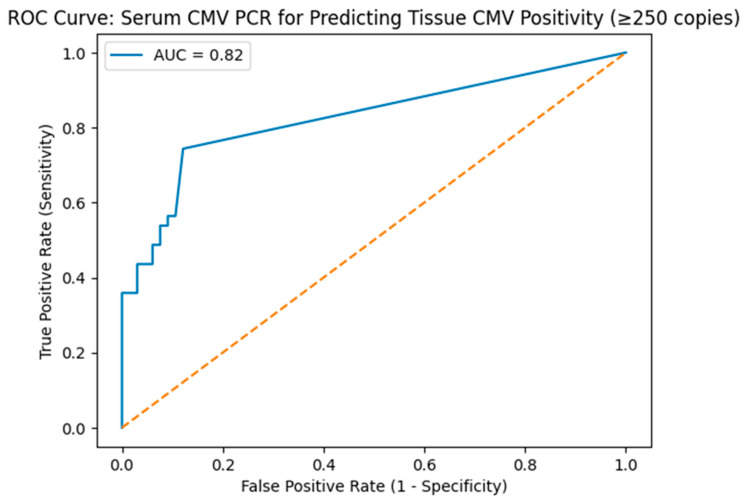
ROC Curve: Serum CMV PCR for Predicting Tissue CMV Positivity. The blue line represents the ROC curve of the serum CMV PCR model for predicting tissue CMV DNA positivity. Orange line represents no-discrimination line (random classifier, AUC = 0.5). Receiver operating characteristic (ROC) curve of serum CMV PCR for predicting tissue CMV DNA positivity (≥250 copies). The area under the curve (AUC) was 0.82, indicating good discriminatory ability. The diagonal dashed line represents random classification.

Finally, the diagnostic performance of serum CMV PCR for predicting tissue CMV DNA positivity (≥250 copies) was evaluated using receiver operating characteristic (ROC) curve analysis (Table 6, Figure 2). Serum CMV PCR demonstrated good discriminatory ability, with an area under the curve (AUC) of 0.82. The optimal cut-off value identified using the Youden index was 53 copies, yielding a sensitivity of 55.6% and a specificity of 87.0%, indicating that while serum CMV PCR has limited sensitivity, a positive serum result is highly specific for tissue CMV DNA positivity.

## 4. Discussion

The association between CMV reactivation and IBD remains a subject of ongoing debate. CMV is frequently detected in patients with ulcerative colitis, particularly in those with moderate-to-severe disease or steroid-refractory flares, and its presence has been variably interpreted as either a pathogenic contributor or an epiphenomenon of severe mucosal inflammation. A major source of this controversy lies in differences in diagnostic approaches, as studies relying on histopathology, immunohistochemistry, or molecular techniques often report divergent prevalence rates and clinical implications. In recent years, tissue polymerase chain reaction (PCR) has emerged as a highly sensitive method for detecting CMV DNA; however, the clinical significance of PCR-based CMV detection in the absence of classical histopathological features remains uncertain [7,15,20]. While some investigators regard CMV reactivation as an innocent bystander in severe colitis, others have suggested that even subclinical viral reactivation may contribute to mucosal injury and disease refractoriness [21]. These uncertainties are further compounded by the retrospective nature of most available studies and the lack of standardized thresholds for defining clinically relevant CMV DNA positivity [18,19,20].

Within this context, the present study was designed to specifically evaluate the relationship between tissue CMV DNA positivity, as defined by a quantitative PCR threshold, and endoscopic disease activity in patients with moderate-to-severe ulcerative colitis, independently of classical histopathological CMV colitis.

CMV colitis is rare in patients with Crohn’s disease or mild-to-moderate ulcerative colitis. In patients with severe and/or steroid-refractory ulcerative colitis, local reactivation of CMV can be detected in approximately 21–36% of cases in actively inflamed colon tissue [11,22,23,24]. Also CMV infection may represent a significant factor in the development of therapeutic resistance in IBD, particularly in cases of severe UC [25]. In our study, consistent with the existing literature, tissue CMV DNA positivity was detected in 37% of moderate-to-severe UC patients based on samples obtained with endoscopic procedures.

In patients with IBD, mucosal damage can compromise the intestinal mechanical barrier, potentially predisposing them to viral infections [26]. Consequently, these patients may exhibit a heightened systemic inflammatory response. It is established that patients with severe ulcerative colitis demonstrate elevated laboratory parameters indicative of an inflammatory response compared to those with inactive disease [27]. Furthermore, some studies indicate that in endoscopically active ulcerative colitis, the presence of CMV in tissue samples is associated with higher levels of inflammatory response parameters such as Erythrocyte Sedimentation Rate ESR and CRP compared to those without detectable CMV [2,22,28]. In the present study, although patients with tissue CMV DNA positivity exhibited higher endoscopic and clinical disease activity, no statistically significant differences were observed in systemic inflammatory markers, including WBC count, CRP, or ESR, between CMV-positive and CMV-negative groups. This finding suggests that the association between tissue CMV detection and disease severity may be more closely related to local mucosal inflammation rather than systemic inflammatory burden. The similarity in acute-phase reactants at the time of patient enrollment may reflect the homogeneous inclusion of patients during active disease flares.

The endoscopic evaluation of mucosal damage plays a crucial role in assessing the severity of UC. While both established and novel biomarkers, including fecal calprotectin and histopathological scoring, offer supplementary insights into biological disease activity, endoscopy remains the gold standard for an objective evaluation of the disease [29]. Consequently, routine endoscopic procedures for evaluating disease status are advocated in the clinical management guidelines by the American College of Gastroenterology, the international consensus statement known as STRIDE (Selecting Therapeutic Targets in Inflammatory Bowel Disease), and by regulatory authorities within the context of clinical trials [30]. The primary objective of our study was to investigate the relationship between the presence and copy number of CMV, detected by tissue rtPCR, and the degree of endoscopic activity in patients with moderate to severe UC. To achieve this aim, we employed the Mayo and Rachmilewitz scoring systems, which have been in use for nearly three decades and are well familiar to clinicians [29]. In our cohort, patients with tissue CMV DNA positivity exhibited significantly higher Mayo clinical scores and Rachmilewitz Endoscopic Activity Index scores compared with CMV-negative patients. However, when tissue CMV DNA copy number was analyzed as a continuous variable, no significant linear correlation was observed with Mayo clinical or Mayo endoscopic scores, and only a borderline association was noted with the Rachmilewitz Endoscopic Activity Index. These findings suggest that the presence of tissue CMV DNA above a defined threshold, rather than the absolute viral copy number, is more closely associated with increased endoscopic disease activity. On the other hand, to understand whether high activity indices serve as indicators of tissue CMV positivity, logistic regression analysis was performed; however, it was observed that neither the elevation of the Mayo score nor the Rachmilewitz score had a significant effect on predicting tissue CMV positivity. The literature contains a limited number of studies examining CMV positivity and endoscopic findings during ulcerative colitis flare-ups [31,32,33]. Suzuki et al. reported that the sensitivity of irregular ulceration for detecting positive CMV was 100%, while the specificity of wide mucosal defects was 95%. Additionally, punched-out and longitudinal ulcerations demonstrated relatively high sensitivity and specificity, exceeding 70% for each, and Yang et al. also reported similar findings [31,33]. To our knowledge, few studies have directly examined the relationship between tissue CMV DNA positivity assessed by PCR and quantitative endoscopic activity scores. A study involving 25 patients with tissue CMV PCR positivity reported endoscopic activity scores before and after antiviral treatment. In that study, the median Rachmilewitz score prior to treatment was 10 (7–12), which is comparable to the scores observed in our CMV-positive cohort. Notably, no significant difference was observed between endoscopic activity scores during the flare and after treatment in those patients [6].

Cytomegalovirus (CMV) infection has been implicated as a contributing factor in the relapse of inflammatory bowel disease [33]. This association has been predominantly noted in patients with ulcerative colitis, particularly among those receiving high-dose corticosteroid therapy [34]. In our study, multivariable logistic regression analysis demonstrated no significant association between anti-TNF therapy and tissue CMV PCR positivity. In contrast, corticosteroid use within the preceding three months was independently associated with tissue CMV DNA positivity, consistent with a moderate increase in risk rather than a marked effect size.

In contemporary medical research, precise diagnostic criteria for confirming CMV infection in UC, particularly in immunosuppressed patients, remain an evolving area, and universally accepted thresholds for tissue CMV DNA copy numbers have not yet been established [19]. In this study, we specifically evaluated whether molecular detection of CMV by tissue PCR is associated with endoscopic disease activity, even in the absence of classical histopathological evidence of CMV infection. Tissue CMV DNA positivity was defined using a threshold of ≥250 copies, based on previous studies suggesting that this level may reflect clinically relevant viral replication rather than latent viral presence [19]. To further address the lack of standardized cut-off values, we performed sensitivity analyses using multiple tissue CMV DNA thresholds (≥100, ≥250, ≥500, and ≥1000 copies). These analyses did not demonstrate a consistent dose–response relationship between increasing viral load and endoscopic disease activity, supporting the use of a pragmatic threshold-based definition rather than reliance on absolute copy number.

Notably, among the 41 patients who met this molecular criterion, only three demonstrated histopathological or immunohistochemical features consistent with classical CMV infection. This finding highlights a critical diagnostic challenge, as some guidelines and studies consider tissue CMV DNA copy numbers of ≥250, when interpreted alongside clinical findings, to be sufficient for diagnosis, whereas other authorities continue to prioritize histopathological confirmation as the diagnostic gold standard [35,36,37]. To accurately reflect this nuance, we deliberately used the term “patients with tissue CMV DNA positivity” rather than “patients with CMV infection” throughout our analysis. Accordingly, antiviral therapy was reserved exclusively for patients with confirmed histopathological or immunohistochemical evidence of CMV, while standard management strategies for UC flares were applied in cases with elevated CMV DNA copy numbers but lacking definitive histological features. By adopting this approach and by applying a predefined molecular threshold supported by sensitivity analyses, we aimed to minimize the risk of overdiagnosis while preserving the ability to detect clinically relevant CMV reactivation. This approach allowed us to specifically assess the association between molecular CMV detection and endoscopic activity without overestimating the clinical significance of subclinical viral presence. In this context, future studies integrating clinical, endoscopic, laboratory, and molecular data using artificial intelligence–based approaches may help to better delineate clinically meaningful CMV reactivation and support more individualized diagnostic and therapeutic decision-making in ulcerative colitis [38].

### Limitations

This study has several limitations that should be considered when interpreting the results. First, its retrospective, single-center design limits the ability to establish causal relationships and may reduce the generalizability of the findings to broader ulcerative colitis populations. Although we focused on a relatively homogeneous group of patients with moderate-to-severe disease, residual confounding—particularly related to underlying disease severity and treatment history—cannot be fully excluded. Second, the sample size was modest, which may have limited the statistical power of certain analyses. This limitation is particularly relevant for the multivariable logistic regression models, in which the association between recent steroid use and tissue CMV DNA positivity was accompanied by relatively wide confidence intervals, suggesting potential model instability. Accordingly, these findings should be interpreted as hypothesis-generating rather than definitive. Third, endoscopic activity scores were assessed using validated scoring systems; however, these evaluations inherently rely on endoscopist interpretation. Although experienced gastroenterologists performed all procedures, endoscopists were not blinded to the clinical context, which may have introduced observer bias. Fourth, although we performed additional sensitivity analyses using multiple tissue CMV DNA thresholds (≥100, ≥250, ≥500, and ≥1000 copies), these analyses were limited by small subgroup sizes at higher viral load cut-offs, precluding robust conclusions regarding very high CMV DNA levels. Similarly, the diagnostic performance of serum CMV PCR was evaluated using ROC analysis; however, serum CMV PCR measurements were not available for all patients, and the retrospective design limited the assessment of serial viral load changes over time.

Finally, the retrospective nature of the study precluded longitudinal evaluation of dynamic changes in tissue and serum CMV DNA levels in relation to treatment response and disease course. Prospective, multicenter studies with standardized diagnostic criteria, predefined viral load thresholds, and longitudinal follow-up are needed to better clarify the clinical significance of tissue CMV DNA detection in ulcerative colitis.

## 5. Conclusions

This study demonstrates that tissue CMV DNA positivity (≥250 copies) is associated with higher endoscopic and clinical disease activity in patients with moderate-to-severe ulcerative colitis, even in the absence of classical histopathological evidence of CMV infection. Patients with tissue CMV DNA positivity exhibited significantly higher Mayo clinical scores and Rachmilewitz Endoscopic Activity Index scores, as well as markedly increased serum CMV DNA levels, compared with CMV-negative patients. In contrast, systemic inflammatory markers did not differ significantly between groups, suggesting that the observed association between CMV detection and disease severity is primarily related to local mucosal activity rather than systemic inflammation. In addition, recent corticosteroid exposure was independently associated with tissue CMV DNA positivity.

These findings suggest that molecular detection of CMV in colonic tissue may provide clinically relevant information in selected patient populations, particularly in patients with moderate-to-severe ulcerative colitis and recent steroid use. However, given the retrospective design and single-center nature of this study, tissue CMV DNA positivity should be interpreted as a molecular association rather than definitive evidence of causality or an indication for antiviral therapy in the absence of histopathological confirmation. Larger, multicenter prospective studies with standardized diagnostic criteria and longitudinal follow-up are warranted to further clarify the clinical significance of tissue CMV DNA detection and its potential role in guiding management strategies in ulcerative colitis.

## Figures and Tables

**Table 1:** Modified Mayo Score and Rachmilewitz Endoscopic Activity Index.

**Modified Mayo Score**				
Score	0	1	2	3
Stool frequency	Normal	1–2 more than normal	3–4 more than normal	4–5 more than normal
Rectal bleed	No bleed	Streaks of blood with stool less than half the time	Obvious blood most of the time	Blood alone
Endoscopic findings	Normal	Mild	Moderate	Severe
Physician Global Assessment	Normal	Mild	Moderate	Severe
**Rachmilewitz Endoscopic Activity Index**				
Score	0	2	4	
Granulation scattering reflected light	No	Yes		
Vascular appearance	Normal	Faded/disturbed	Completely absent	
Fragility of mucosa	None	Contact bleeding	Spontaneous bleeding	
Mucosal damage (mucus, fibrin, exudate, erosion, ulcer)	None	Slight	Pronounced	

Modified Mayo Score: Clinical disease activity measured across stool frequency, rectal bleeding, endoscopic findings, and physician global assessment (each subscore 0–3; higher totals indicate greater disease severity). Rachmilewitz Endoscopic Activity Index: Composite endoscopic score of granulation, vascular appearance, mucosal fragility and damage; higher values reflect greater inflammation.

**Table 2:** Comparison of some parameters between CMV+ and CMV− active ulcerative colitis patients.

Variable	CMV Tissue (+) ≥ 250 Copies (*n* = 41)	CMV Tissue (–) < 250 Copies (*n* = 69)	*p* Value
Age, years	48.7 ± 20.4; median 54 (30–62)	43.1 ± 16.3; median 43 (29–55)	0.148
Female sex, *n* (%)	19 (46.3%)	23 (33.3%)	0.248
Follow-up time, months	63.1 ± 53.2; median 60 (18–72)	84.6 ± 71.5; median 66 (24–122.5)	0.173
WBC (×10^3^/µL)	10,310.9 ± 11,036.4; median 7330 (6080–9500)	9480.1 ± 4143.7; median 8900 (6230–12,100)	0.296
CRP (mg/L)	45.3 ± 57.8; median 22 (5–64)	31.1 ± 46.0; median 8 (4–40)	0.186
ESR (mm/h)	52.2 ± 22.3; median 54 (36.5–67)	48.7 ± 29.3; median 44 (29–64)	0.188
Daily defecation frequency (*n*/day)	10.8 ± 5.9; median 10 (6–15)	10.5 ± 6.9; median 10 (5–15)	0.558
Serum CMV PCR (copies)	1104.3 ± 2520.7; median 57 (21–1006)	18.3 ± 66.5; median 0 (0–0)	<0.001
Rachmilewitz Endoscopic Activity Index	9.0 ± 2.3; median 8 (8–10)	7.8 ± 2.5; median 8 (6–10)	0.021
Mayo clinical score	8.7 ± 2.5; median 10 (8–11)	7.6 ± 2.4; median 8 (6–10)	0.014
Mayo endoscopic score	2.5 ± 0.5; median 2 (2–3)	2.3 ± 0.6; median 2 (2–3)	0.161

Abbreviations: WBC: white blood cell, ESR: erythrocyte sedimentation rate.

**Table 3:** Correlation analysis between tissue CMV DNA copy number and clinical, laboratory, and endoscopic parameters (Spearman).

Parameter	*n* (Pairwise)	Spearman r	*p* Value
Age, years	109	0.065	0.499
Age at diagnosis, years	108	0.144	0.138
Follow-up time, months	108	−0.160	0.098
WBC	109	−0.170	0.077
CRP	105	0.065	0.510
ESR (sedimentation)	101	0.088	0.380
Daily defecation frequency	104	−0.029	0.769
Serum CMV PCR (copies)	104	0.595	<0.001
Mayo clinical score	100	−0.012	0.908
Mayo endoscopic score	109	0.103	0.288
Rachmilewitz Endoscopic Activity Index	109	0.179	0.063

**Table 4:** Multivariable logistic regression models for predictors of tissue CMV PCR positivity (≥250 copies).

Variable	OR (95% CI)	*p* Value
Model 1. Clinical risk model		
Age (per year)	1.02 (0.99–1.05)	0.178
Female sex	2.00 (0.79–5.07)	0.145
Follow-up time (months)	0.99 (0.98–1.00)	0.087
Steroid use in the last 3 months	2.58 (1.01–6.62)	0.048
Anti-TNF therapy	1.08 (0.36–3.27)	0.893
Model 1 statistics	Pseudo R^2^ = 0.082	LRT *p* = 0.067
Model 2. Disease activity–adjusted model		
Female sex	1.98 (0.80–4.91)	0.140
Follow-up time (months)	0.99 (0.99–1.00)	0.144
Steroid use in the last 3 months	2.32 (0.92–5.85)	0.076
Rachmilewitz Endoscopic Activity Index	1.13 (0.93–1.37)	0.211
Model 2 statistics	Pseudo R^2^ = 0.079	LRT *p* = 0.041

**Table 5:** Sensitivity analysis of different tissue CMV DNA thresholds and their association with disease activity.

Tissue CMV DNA Threshold	CMV-Positive (*n*)	Mayo Clinical Score (CMV+)	Mayo Clinical Score (CMV−)	*p* Value	Rachmilewitz EAI (CMV+)	Rachmilewitz EAI (CMV−)	*p* Value
≥100 copies	43	8.6 ± 2.4	8.7 ± 2.3	NS	8.8 ± 1.9	7.9 ± 2.7	0.085
≥250 copies (*primary analysis*)	29	8.6 ± 2.6	8.7 ± 2.3	NS	8.7 ± 2.2	8.1 ± 2.5	NS
≥500 copies	18	8.2 ± 2.7	8.8 ± 2.3	NS	8.3 ± 2.0	8.2 ± 2.5	NS
≥1000 copies	11	8.4 ± 1.7	8.7 ± 2.4	NS	8.1 ± 1.9	8.3 ± 2.5	NS

Abbreviations: CMV: cytomegalovirus; EAI: Endoscopic Activity Index; NS: not significant; CMV+: indicates tissue CMV DNA levels ≥ the specified threshold; CMV−: indicates levels below the respective threshold.

**Table 6:** Diagnostic performance of serum CMV PCR for predicting tissue CMV DNA positivity (≥250 copies).

Parameter	Value
Area under the curve (AUC)	0.82
Optimal cut-off (Youden index)	53 copies
Sensitivity	55.6%
Specificity	87.0%

## Data Availability

The data can be found in the data repository of the Gastroenterology Department at Ondokuz Mayıs University Faculty of Medicine. If you wish to request access to the data, please contact us at abektas@omu.edu.tr or ufukavcioglu@yahoo.com, or please visit https://hastane.omu.edu.tr/?p=1276 (accessed on 10 April 2024).

## Abbreviations

CMV	Cytomegalovirus
UC	Ulcerative colitis
PCR	Polymerase chain reaction (including tissue PCR, serum PCR, RT-PCR)
IHC	Immunohistochemistry
H&E	Haematoxylin and Eosin (staining)
ECCO	European Crohn’s and Colitis Organization
EAI	Endoscopic Activity Index (Rachmilewitz Endoscopic Activity Index)
ESR(Sedim)	Erythrocyte sedimentation rate
CRP	C-reactive protein
WBC	White blood cell (count)
anti-TNF	Anti–tumor necrosis factor (therapy/agent)
STRIDE	Selecting Therapeutic Targets in Inflammatory Bowel Disease (international consensus/treat-to-target)
IBD	Inflammatory bowel disease
RT-PCR/rtPCR	Reverse transcription (or real-time) polymerase chain reaction (context: quantitative tissue CMV detection)
DNA	Deoxyribonucleic acid
GI	Gastrointestinal (used in context of GI tract/Gastrointestinal Endoscopy)
